# Reactive Palatal Fibrosis Following Facial Dermal Filler Injection and Anfiye Inhalation: A Case Mimicking OSMF

**DOI:** 10.1002/ccr3.71803

**Published:** 2026-01-28

**Authors:** Farshad Javadzadeh, Maryam Hoseinpour Sarmadi, Mina Zohrabi

**Affiliations:** ^1^ Department of Oral & Maxillofacial Medicine, Faculty of Dentistry Tabriz University of Medical Sciences Tabriz Iran

**Keywords:** dermal filler, mouth neoplasms, *nigella sativa*, oral submucous fibrosis

## Abstract

Anfiye is a traditional herbal remedy commonly used in Iran and several Arabic countries for the treatment of sinusitis and nasal congestion. It is typically administered as a nasally inhaled powder. Various formulations exist under the name Anfiye; however, the most widely used preparation contains root powder of *Gypsophila struthium* and seeds of *Nigella sativa* . Inhalation of Anfiye induces sneezing, which in turn helps alleviate nasal congestion and rhinorrhea, thereby contributing to the management of sinusitis. Modifying facial soft tissues volume and contours through dermal injection of botulinum toxin, injectable fillers such as hyaluronic acid, and bovine collagen is a common esthetic procedure these days. Although they may be successfully utilized, there may be possible side effects such as allergic reactions, inflammation, foreign body granuloma formation, and skin necrosis due to incorrect injection techniques. Also, it is a relatively expensive esthetic treatment. In this case report, we present a 41‐year‐old Iranian woman who developed swelling in the right portion of the palate following injection of facial dermal filler and use of Anfiye for treating sinusitis. An excisional biopsy was performed, and histopathological analysis revealed marked submucosal fibrosis with features reminiscent of oral submucous fibrosis (OSMF). However, the absence of hallmark clinical criteria—namely palpable fibrotic bands, progressive reduction in tissue elasticity, and trismus or limited mouth opening—alongside the patient's denial of areca nut use and spontaneous healing of the lesion after biopsy suggests that the presentation is more consistent with reactive palatal fibrosis secondary to facial filler injection and Anfiye inhalation. This report aims to highlight Anfiye or dermal fillers as potential etiological factors in the development of reactive fibrosis, which can be misdiagnosed as OSMF.

**Key Clinical Message**

Early detection of OSMF is essential due to its premalignant nature. Any fibrotic white lesion with altered texture should prompt consideration of OSMF. Subtle initial signs require detailed history, histopathology, and thorough oral examination, emphasizing fibrotic bands, reduced elasticity, and mouth opening.

## 1 Introduction

Oral Submucous Fibrosis (OSMF) is a long‐term, gradually worsening condition with malignant potential, impacting the oral cavity, oropharynx, and the upper third of the esophagus. Malignant transformation was first described by Paymaster in 1956, who observed oral squamous cell carcinomas in a third of a cohort affected by the disorder [1, 2]. Males are much more prone to OSMF, which is related to their higher levels of areca nut‐based products use [3]. The disease may onset at any age, but most commonly affects adolescents and adults under the age of 35 [4]. The incidence rate of OSMF exhibits a distinct dose‐dependent correlation with the frequency of areca nut chewing [5].

Anfiye, also called saeut, is a traditional herbal medication used for many disorders, including sinusitis, nasal congestion, and rhinorrhea. The word Anf means nose in Arabic, so every medication inhaled through the nasal route can be generally called Anfiye, but Anfiye is specifically a combination of the root powder of *Gypsophila struthium* and seeds of *Nigella sativa* . As in traditional medicine, sneezing plays an important role in preventing and treating many diseases, including cerebrovascular accident (CVA); medications such as Anfiye, which induce sneezing, are crucial. It has been said that sneezing enhances blood circulation in the head, improves vision, and descales the heart and lungs. After inhaling Anfiye, severe sneezing leads to the removal of harmful brain fluids and discharge of sinuses. Also, it alleviates nasal congestion and rhinorrhea. It is contraindicated in pregnancy, heart failure, patients with recent surgery, and patients who have sutures in their bodies because sneezing can worsen their condition.

The face is an important part of the body, having a close connection with beauty, youth, health, and self‐confidence. Hence, seeking new ways of augmenting facial volume or recontouring it is a critical field in esthetic science and is also crucial and interesting for individuals [6]. Fillers perform this aim by means of physical filling or stimulating new collagen production. The classical fillers include hyaluronic acid (HA) and collagen, with the latter being more effective. Side effects of injecting HA fillers, which have been documented so far, include allergic symptoms such as transient erythema, pruritus, swelling, and more severe ones like foreign body granulomas and vascular occlusion. On the other hand, collagen fillers have more immunogenicity compared to HA fillers, which makes the patient susceptible to viral infections as they have a limited tolerance to terminal sterilization [7].

This report presents a case of reactive palatal fibrosis that developed following facial dermal filler injection and Anfiye inhalation, exhibiting histopathological features mimicking OSMF, yet lacking the clinical manifestations and historical factors typically associated with OSMF.

## 2 Case History/Examination

The patient in our study was a 41‐year‐old Iranian woman with a history of migraine who did not use any medications. She had no history of smoking, alcohol consumption, or areca nut chewing. In November 2023, she experienced severe unilateral pain involving the right side of her face, eye, and hard palate following generalized facial dermal filler injections in multiple areas.

**FIGURE 1:**
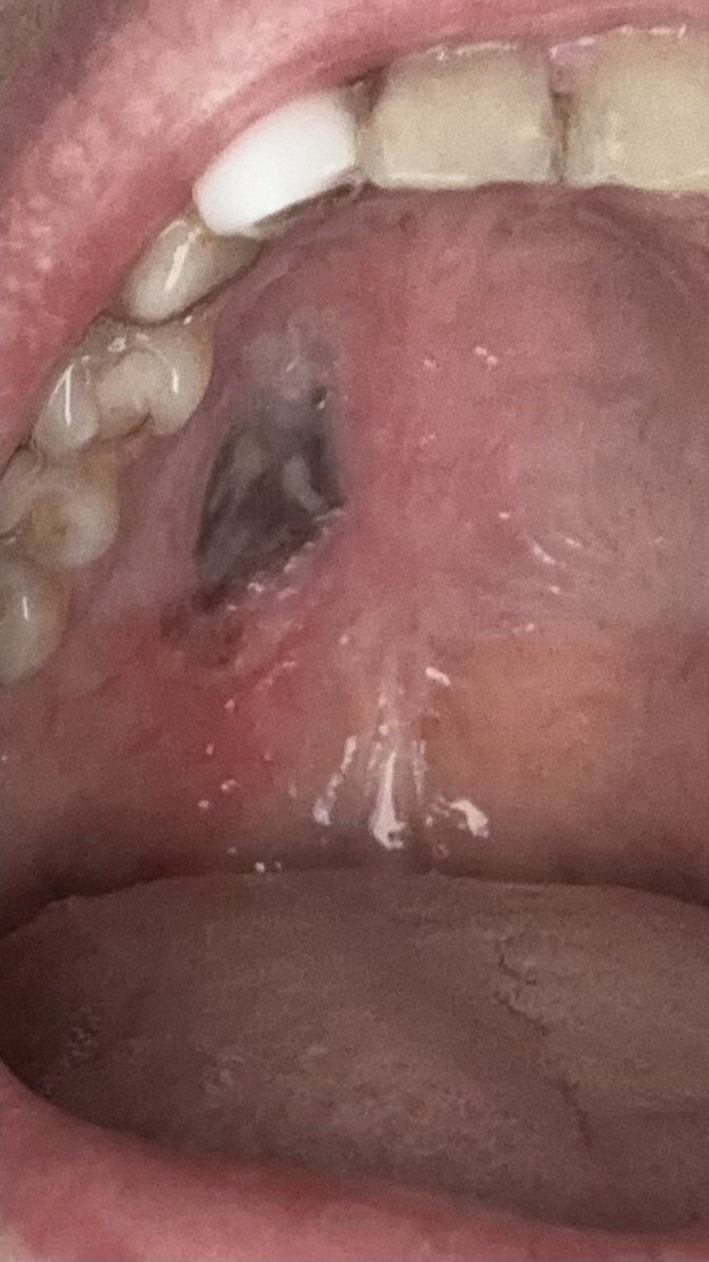
Black ulcer formed after inhaling Anfiye for two months in the right portion of the hard palate post‐dermal filler injection.

After consulting an ophthalmologist and undergoing magnetic resonance imaging (MRI), no identifiable pathology was detected. Suspecting sinusitis as a possible cause of the pain, she was referred to a traditional medicine center, where facial phlebotomy was performed. Additionally, Anfiye was prescribed for two months. As the pain persisted despite these treatments, she sought further medical attention. A severe black ulcer had developed on the right side of her hard palate (Figure 1).

Due to ongoing discomfort, the patient repeatedly stimulated the ulcer with her tongue and reported the discharge of white material from the lesion. One month later, when the ulcer had not fully healed, swelling developed, and the pain continued. She was subsequently referred to an Oral and Maxillofacial Medicine specialist in September 2024.

**FIGURE 2:**
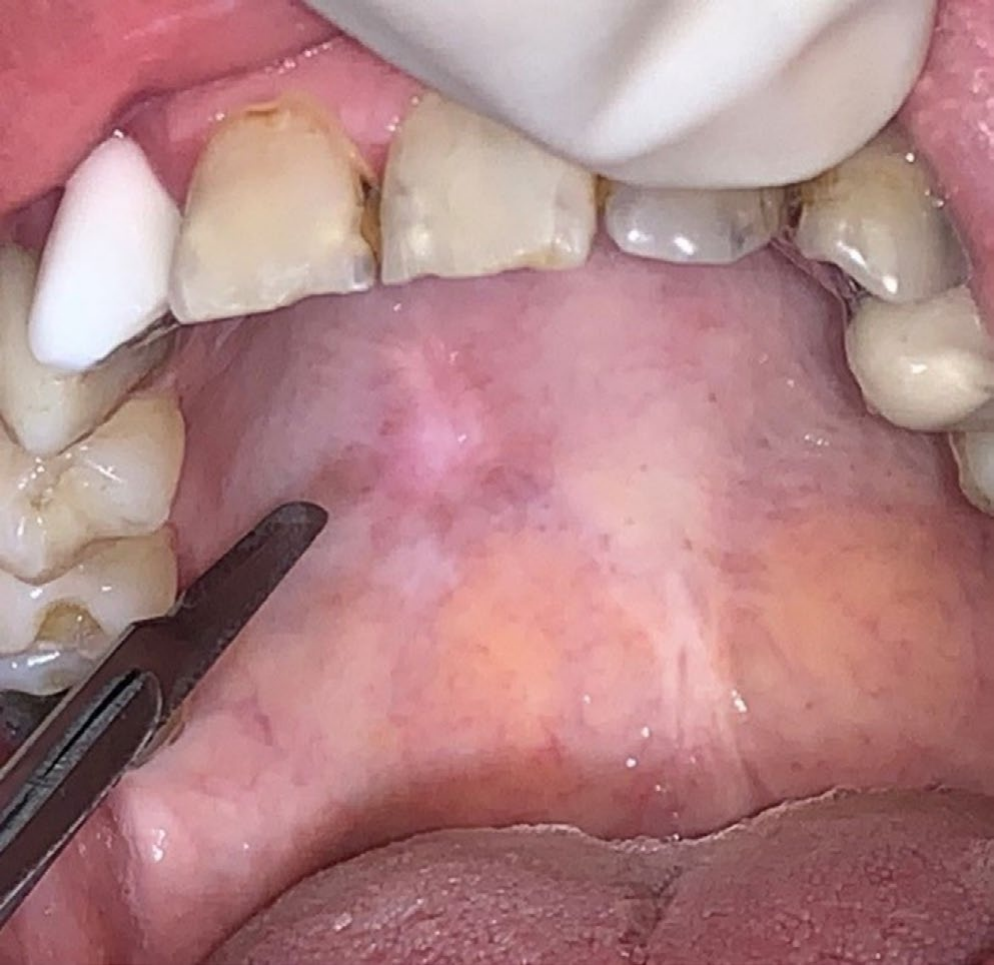
Exophytic nodular lesion with a depressed center and pale color in the right portion of the hard palate with a surrounding blanched area and pigmentation.

Clinical examination revealed an exophytic nodular lesion with a depressed center and pale coloration, with a history of intermittent enlargement and regression (Figure 2). The entire right hard palate mucosa exhibited a white, marbled appearance with blanching, and areas of pigmentation were noted surrounding the nodular lesion. The patient demonstrated no limitation in mouth opening, with a normal maximal interincisal distance. No palpable fibrotic bands or changes in mucosal texture were observed.

## 3 Differential Diagnosis, Investigations, and Treatment

**FIGURE 3:**
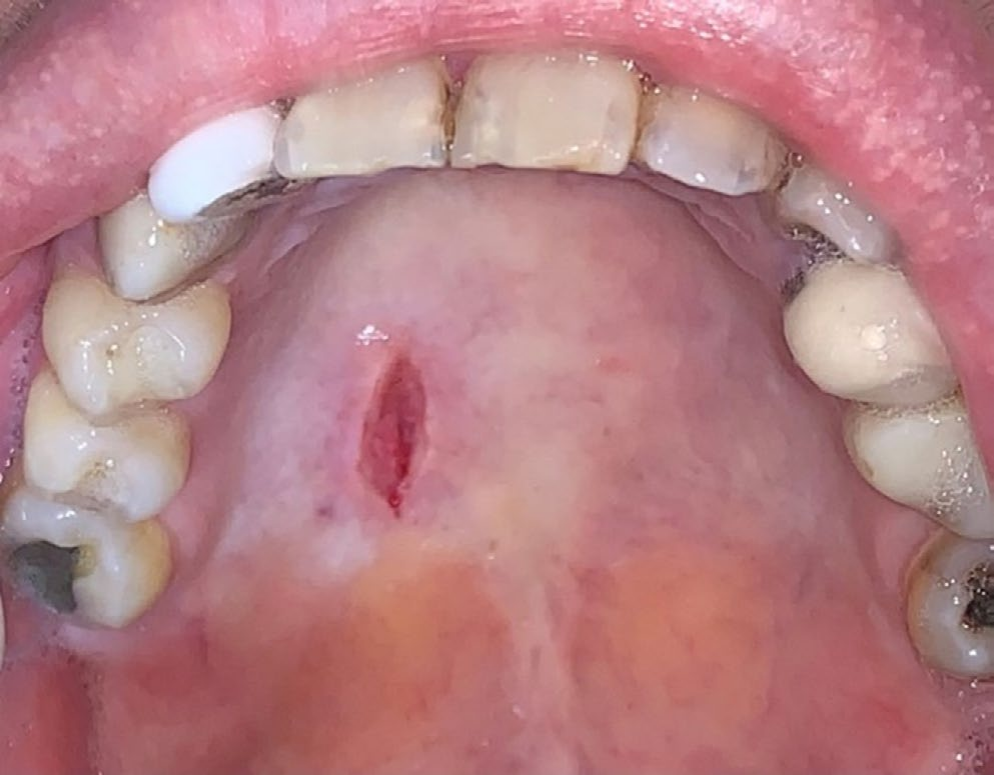
Excisional biopsy taken from the lesion.

An excisional biopsy of the nodular lesion was obtained with differential diagnosis of mucocele, traumatic ulcer, and minor salivary gland tumor (Figure 3).

**FIGURE 4:**
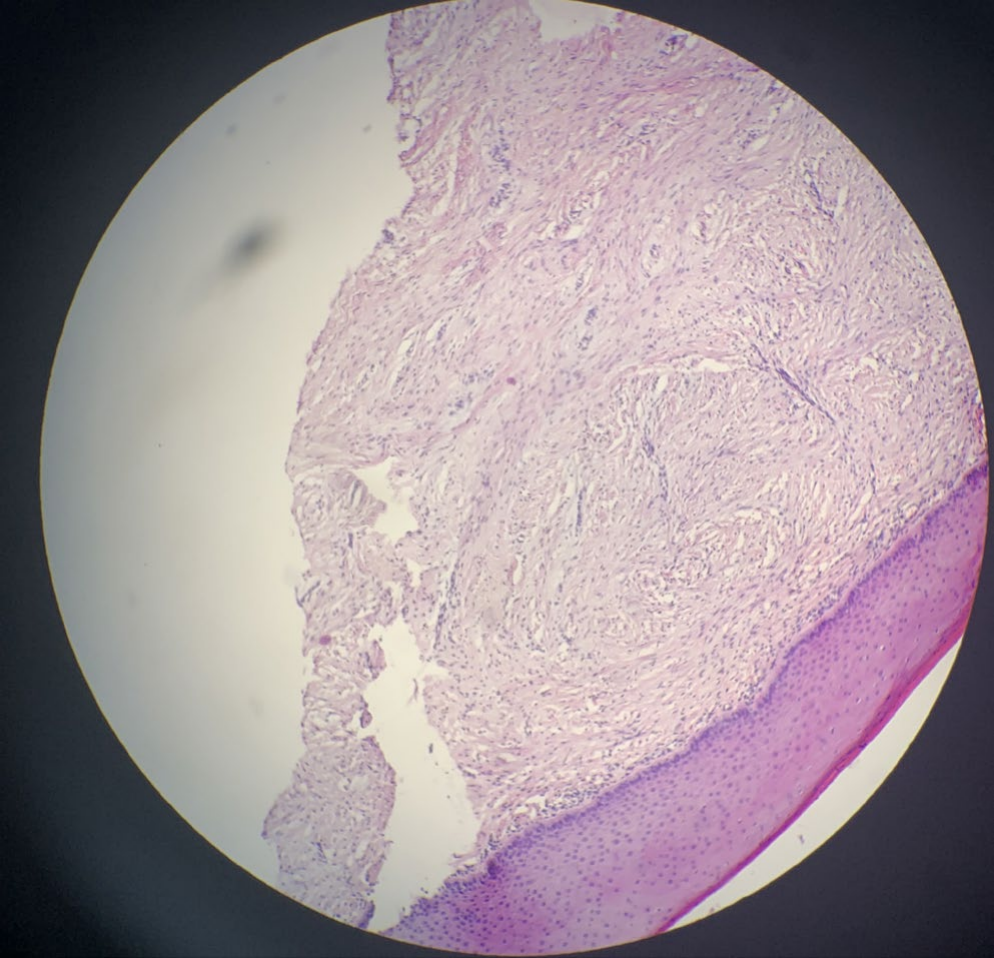
Histopathologic view indicating parakeratotic squamous mucosa with an underlying stroma and superficially mild chronic inflammatory cells and proliferation of fibroblasts (H&E staining, Magnification x4).

Histopathologic view showed squamous mucosa with parakeratosis, an underlying stroma with superficially mild chronic inflammatory cells and proliferation of fibroblasts, and no cystic lesion, nor epithelial tumor or ulceration, suggesting Submucosal Fibrosis (Figure 4).

Diagnosis of OSMF is based on both the presence of at least one of the following characteristics and histopathologic confirmation.

- Palpable fibrous bands
- Progressive loss of tissue elasticity
- Trismus or reduced mouth opening

Furthermore, the development of OSMF in the absence of areca nut consumption is exceedingly uncommon, as areca nut use is recognized as a primary etiological factor in nearly all documented cases. Notably, OSMF is a progressive disorder that does not resolve following conventional biopsy procedures.

Although the histopathological findings in our case were suggestive of OSMF, the absence of supporting clinical signs and relevant history led us to exclude OSMF as the final diagnosis. Instead, the condition was identified as reactive fibrosis, likely induced by facial dermal filler injection and Anfiye inhalation.

As she had no restrictions in function or opening her mouth, and the lesion had resolved completely in the first follow‐up session, we considered excisional biopsy as both a diagnostic workup and treatment. Also, palliative therapy, including vitamin and mineral supplementation, was prescribed.

## 4 Conclusion and Results (Outcome and Follow‐Up)

**FIGURE 5:**
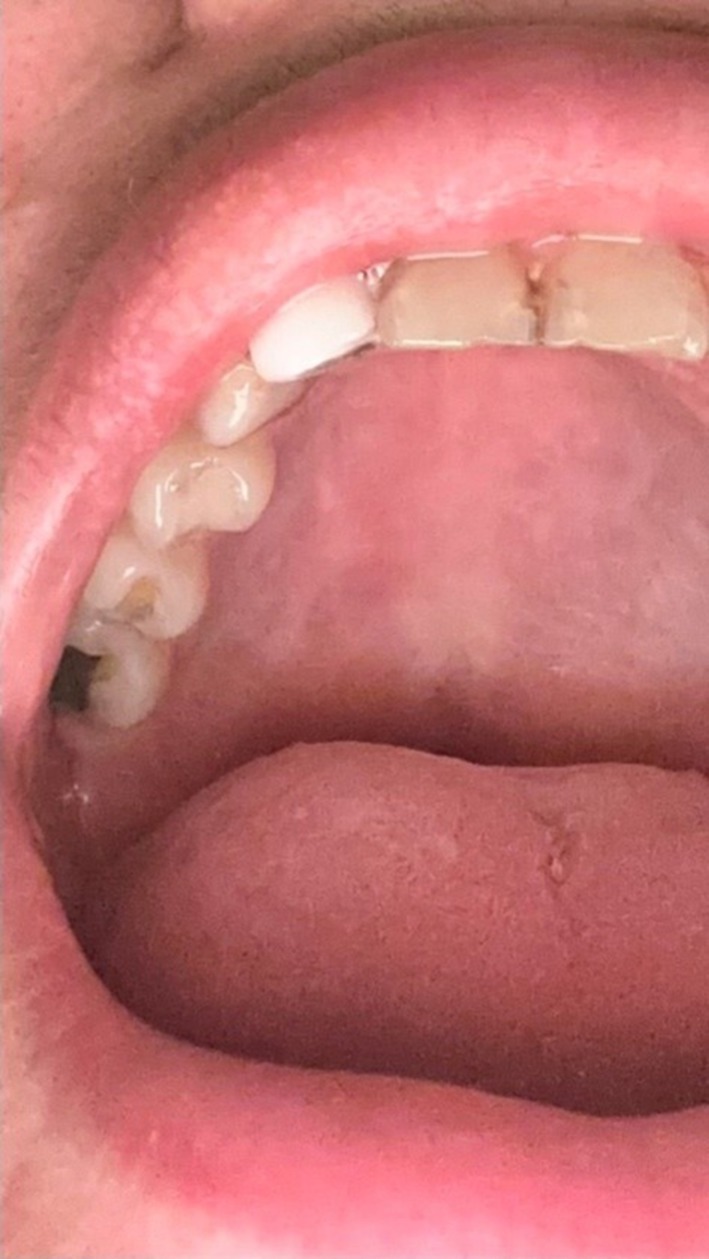
Complete healing of the area after biopsy.

Although the final diagnosis was decided to be reactive fibrosis, not OSMF, the patient was advised to take follow‐up sessions seriously and to self‐examine for any changes in the texture, color, and structure of her whole oral components. Unfortunately, after the second follow‐up, which was done in November 2024, she migrated to another country, and we lost close access to her. However, in these follow‐up sessions, a full examination and toluidine‐blue staining were done, but there was no evidence of active lesion or dysplasia, and the area of biopsy had healed normally (Figure 5). She was advised to visit an Oral and Maxillofacial Medicine specialist every 3 months through the first year, and every 6 months after in her new living place. On the last contact with her, there was no report of recurrence, but the patient still had a mild unilateral pain in the right portion of the face, eye, and hard palate.

Although histopathologic features of fibrosis‐related disorders may overlap, taking a complete history of risk factors, paying attention to signs and symptoms can help prevent misdiagnosis. Hence, the presence of clinical features such as inelastic, white, and blanching mucosa or changes in surface texture with a history of pain and burning or mouth opening limitations and areca nut chewing, along with confirmatory histopathology, is necessary for a definite diagnosis of OSMF.

## 5 Discussion

Etiopathogenesis of OSMF is believed to be a kind of disorder in the metabolism of collagen, an excessive repair process that occurs after persistent chronic injury [1, 8]. Conducted studies illuminate the elevated production of subepithelial collagen and reduced collagen degeneration in the extracellular matrix (ECM) as the principal etiology, influencing mouth movements [9, 10]. OSMF has a multifactorial nature engaging both environmental factors (capsaicin in chilies, tobacco, copper toxicity, vitamin deficiency, malnutrition, and anemia) and genetics, as well as immunological contributors. There is general agreement that the consumption of areca nut is the leading cause of OSMF formation [3, 9]. OSMF affects all three types of masticatory, lining, and specialized mucosa, with most commonly involving the buccal mucosa [9, 10], retromolar area, and the soft palate region [10]. Patients often recall specialists with a chief complaint of pain and burning sensation of the oral mucosa, followed by ulcers, blisters, a diminished taste sensation, xerostomia, lip and tongue paresthesia, and other symptoms, including a profound hardship in mouth opening, dysphagia, and impaired tongue movement [1]. Oral mucosal whitening is a key feature in the initial phases in which the soft and pink normal oral mucosa develops to a fibrotic and pale tissue, demonstrating papery white and tough on palpation, with a dense vertical band. Also, the presence of erythema, petechiae, pigmentation, or vesicles can be the first signs of the disease. Restriction in mouth opening caused by fibrosis of the oral mucosa is a characteristic feature of advanced disease [10]. OSMF has a significant correlation to oral carcinoma, with a marked malignant transformation potential of 7%–30% [11]. Notably, initial OSMF lesions also have a high tendency to develop into malignancy [12]. Two main treatment procedures can be conducted: Nonsurgical and surgical therapy. Corticosteroids, anti‐inflammatory cytokines, such as interferon‐gamma (IFN‐γ), ameliorate inflammation and reduce collagen production and storage. Previous studies have demonstrated that enzymes such as hyaluronidase, collagenase, and chymotrypsin have been employed to inhibit ECM deposition, a strategy that has long been considered effective in the management of OSMF. Supplementary medications, such as vitamins, minerals, and vasodilators, are utilized for reducing symptoms and improving burning sensation and ulcers [8, 10]. Indication of Surgery is limited to severe OSMF, when the maximum mouth opening fails to meet 20 mm. Scalpel blades, electrocautery, and laser therapy are recommended surgical modalities [13].

Dermal fillers (DF) rank among the most frequently performed dermatologic procedures, with their utilization steadily increasing each year. Common DF materials include hyaluronic acid (HA), calcium hydroxylapatite, poly‐L‐lactic acid, silicone, and polymethylmethacrylate (PMMA). Although there is no universally accepted timeline distinguishing early from late adverse reactions, the World Health Organization (WHO) classifies mild adverse events—such as localized swelling, bruising, and erythema at the injection site—as typically self‐limiting and not requiring substantial medical intervention. In contrast, moderate complications, including mycobacterial infections and granulomatous inflammatory responses, often necessitate clinical management [14]. These reactions arise from multiple contributing factors, including the chemical composition of the injected substance, the technique of administration, the cumulative antigenic burden, and, most critically, the host's immunological profile [15]. Fillers can occasionally trigger dermatitis‐like symptoms as a result of allergic reactions to specific components such as collagen, lidocaine, and carboxymethylcellulose (CMC) [16]. Reactive fibrosis has been reported as a consequence of HA fillers injected in the nasolabial fold [17]. Foreign body reactions have also been documented following facial injections with poly‐L‐lactic acid [18]. Therefore, in patients presenting with fibrosis‐related lesions and a history of dermal filler injections, clinicians should consider granulomatous inflammatory reactions as a potential underlying etiology.

Both *Gypsophila* and *Nigella sativa* have demonstrated promising therapeutic effects against pulmonary diseases [19, 20], suggesting their potential efficiency in managing sinusitis. There is no direct evidence supporting their role in inducing fibrosis; however, they may have contributed indirectly through negative pressure generated during episodes of sneezing.

In conclusion, the reactive palatal fibrosis observed in our case may be attributed to one of several etiologies: A granulomatous inflammatory response following dermal filler injection, a contact hypersensitivity reaction triggered by Anfiye inhalation, or post‐traumatic ischemic necrosis resulting from chronic mechanical stimulation by the tongue. Given the absence of areca nut chewing history, lack of classic clinical features of OSMF, and spontaneous healing of the lesion following biopsy, it is more appropriate to refrain from diagnosing this case as OSMF, despite histopathologic similarities.

Notably, there are no documented cases of reactive fibrosis associated with the use of *Gypsophila and Nigella sativa* in combination with facial dermal fillers, which highlights the novelty of our study. Further studies, including animal studies and retrospective cohort studies, are needed for more accurate data about the possibility of Anfiye's components and dermal fillers to induce submucosal fibrosis individually or in combination.

## Author Contributions

**Farshad Javadzadeh:** data curation, investigation, methodology, supervision, writing – review and editing. **Maryam Hoseinpour Sarmadi:** conceptualization, project administration, validation, visualization, writing – review and editing. **Mina Zohrabi:** investigation, methodology, resources, writing – original draft, writing – review and editing.

## Funding

The authors have nothing to report.

## Consent

Written informed consent was obtained from the patient to publish this report in accordance with the journal's patient consent policy.

## Conflicts of Interest

The authors declare no conflicts of interest.

## Data Availability

The data that support the findings of this study are available in this article.

## References

[1] P. Y. Chen , S. C. Chao , P. L. Hsieh , et al., “Butylidenephthalide Abrogates the Snail‐Induced Cancer Stemness in Oral Carcinomas,” International Journal of Molecular Sciences 23, no. 11 (2022): 6157. 35682836 10.3390/ijms23116157 PMC9180956

[2] J. C. Paymaster , “Cancer of the Buccal Mucosa; a Clinical Study of 650 Cases in Indian Patients,” Cancer 9, no. 3 (1956): 431–435. 13329991 10.1002/1097-0142(195605/06)9:3<431::aid-cncr2820090302>3.0.co;2-t

[3] V. Murthy , P. Mylonas , B. Carey , et al., “Malignant Transformation Rate of Oral Submucous Fibrosis: A Systematic Review and Meta‐Analysis,” Journal of Clinical Medicine 11, no. 7 (2022): 1793, 10.3390/jcm11071793. 35407401 PMC8999767

[4] A. G. Singh , S. Roy , S. Oza , et al., “A Contemporary Narrative Review to Guide Molecular Epidemiology of Oral Submucous Fibrosis,” International Journal of Molecular Epidemiology and Genetics 12, no. 2 (2021): 61–70. 34552689 PMC8449189

[5] Z. Xu , F. Y. Lü , E. H. Jiang , X. P. Zhao , and Z. J. Shang , “Relationship Among Areca Nut, Intracellular Reactive Oxygen Species, and Autophagy,” Hua Xi Kou Qiang Yi Xue Za Zhi = West China Journal of Stomatology 38, no. 1 (2020): 80–85, 10.7518/hxkq.2020.01.014. 32037771 PMC7184295

[6] B. J. Mahmood Faris , “The Use of Facial Fillers in Clinical Practice: The Level of Patient Satisfaction and an Overview of Common Clinical Complications,” Actas Dermo‐Sifiliográficas 115, no. 5 (2024): 458–465, 10.1016/j.ad.2023.10.008 Available from, https://www.sciencedirect.com/science/article/pii/S0001731023008207. 37865230

[7] J. Guo , W. Fang , and F. Wang , “Injectable Fillers: Current Status, Physicochemical Properties, Function Mechanism, and Perspectives,” RSC Advances 13 (2023): 23841–23858, 10.1039/d3ra04321e. 37577103 PMC10413051

[8] J. Tang , J. Liu , Z. Zhou , et al., “Oral Submucous Fibrosis: Pathogenesis and Therapeutic Approaches,” International Journal of Oral Science 17 (2025): 8, 10.1038/s41368-024-00344-6. 39890798 PMC11785813

[9] H. Xu , F. Y. Lyu , J. Y. Song , et al., “Research Achievements of Oral Submucous Fibrosis: Progress and Prospect,” BioMed Research International 2021 (2021): 6631856, 10.1155/2021/6631856. 33791368 PMC7997751

[10] Y. W. Shen , Y. H. Shih , L. J. Fuh , and T. M. Shieh , “Oral Submucous Fibrosis: A Review on Biomarkers, Pathogenic Mechanisms, and Treatments,” International Journal of Molecular Sciences 21, no. 19 (2020): 7231, 10.3390/ijms21197231. 33008091 PMC7582467

[11] N. R. Rao , A. Villa , C. B. More , R. D. Jayasinghe , A. R. Kerr , and N. W. Johnson , “Oral Submucous Fibrosis: A Contemporary Narrative Review With a Proposed Inter‐Professional Approach for an Early Diagnosis and Clinical Management,” Journal of Otolaryngology ‐ Head & Neck Surgery 49, no. 1 (2020): 3, 31915073, 10.1186/s40463-020-0399-7. 31915073 PMC6951010

[12] S. Meera , R. Sarangarajan , and K. Rajkumar , “8‐Isoprostane: A Salivary Oxidative Stress Biomarker for Oral Submucous Fibrosis and Oral Squamous Cell Carcinoma,” Journal of Oral Maxillofac Pathol 24, no. 2 (2020): 279–284, 10.4103/jomfp.JOMFP_235_19. 33456237 PMC7802855

[13] M. Kapre and K. Sudhanshu , Surgery of Trismus in Oral Submucous Fibrosis (Springer), 2018, Available at, https://www.springer.com/gp/book/9789811048906.

[14] P. Gamez‐Siller , H. Moreno‐Davila , M. Oscherwitz , R. Franco‐Marquez , D. A. Galarza‐Delgado , and J. A. Cardenas‐de la Garza , “Granulomatous Filler Reaction Treated With Adalimumab: A Case Report and Literature Review,” Anais Brasileiros de Dermatologia 100, no. 6 (2025): 501214, 10.1016/j.abd.2025.501214. 41177077 PMC12616106

[15] M. G. Tafur and C. Rodríguez‐Cerdeira , “Granulomatous Reactions Following the Injection of Multiple Aesthetic Microimplants: A Complication Associated With Excessive Filler Exposure in a Predisposed Patient,” Report 8, no. 4 (2025): 194, 10.3390/reports8040194. PMC12643430 41133535

[16] G. W. Hong , H. Hu , K. Chang , et al., “Review of the Adverse Effects Associated With Dermal Filler Treatments: Part I Nodules, Granuloma, and Migration,” Diagnostics 14, no. 15 (2024): 1640, 39125515, 10.3390/diagnostics14151640. 39125515 PMC11311355

[17] N. P. Mikhailova , D. I. Znatdinov , M. A. Petriy , and I. V. Borzova , “Hyaluronic Acid in the Improvement of Skin Aging: Optimal Physicochemical Characteristics, Modifications, and Personalized Strategies,” Medical Alphabet 23, no. 1 (2025): 108–112, 10.33667/2078-5631-2025-23-108-112.

[18] Y. J. Jeon , D. W. Koo , and J. S. Lee , “Late Onset Foreign Body Reaction due to Poly‐L‐Lactic Acid Facial Injections for Cosmetic Purpose,” Annals of Dermatology 32, no. 6 (2020): 519, 33911797–522, 10.5021/ad.2020.32.6.519. 33911797 PMC7875245

[19] M. Kamali , M. Talebi , J. Mottaghipisheh , E. Sasani , and B. M. Mirshekari , “An Updated Overview of Gypsophila Species: Phytochemical and Pharmacological Investigations,” Fitoterapia 179 (2024): 106230, 10.1016/j.fitote.2024.106230. 39326798

[20] A. Ahmad , A. Husain , M. Mujeeb , et al., “A Review on Therapeutic Potential of *Nigella Sativa*: A Miracle Herb,” Asian Pacific Journal of Tropical Biomedicine 3, no. 5 (2013): 337, 23646296–352, 10.1016/S2221-1691(13)60075-1. 23646296 PMC3642442

